# Potential impact of land‐use change on habitat quality in the distribution range of crocodile lizards in China

**DOI:** 10.1002/ece3.9390

**Published:** 2022-10-17

**Authors:** Xiaoli Zhang, Xudong Qin, Facundo Alvarez, Zening Chen, Zhengjun Wu

**Affiliations:** ^1^ Key Laboratory of Ecology of Rare and Endangered Species and Environmental Protection (Guangxi Normal University), Ministry of Education Guilin China; ^2^ Present address: Guangxi Key Laboratory of Rare and Endangered Animal Ecology Guangxi Normal University Guilin China; ^3^ Present address: Guangxi Daguishan Crocodile Lizard National Nature Reserve Hezhou China; ^4^ Programa de Pós‐graduação em Ecologia e Conservação, Campus Nova Xavantina Universidade do Estado de Mato Grosso Brazil

**Keywords:** environment landscape, habitat quality, InVEST, *Shinisaurus crocodilurus*, space–time evolution

## Abstract

The over‐exploitation of land resources poses a serious threat to biodiversity on a global scale. Changes in land‐use and human exploitation have had a major impact on wild populations and their habitat in China. We assessed how habitat quality has changed over time (1995–2020). Specifically, we analyzed how the habitat quality of crocodile lizard has changed over time based on multi‐temporal land‐use data (1995, 2000, 2010, 2015 and 2020) using a land‐use transfer matrix and habitat quality model. The results showed that the main landscape types in the study area were arable land (21.21% of the area) and woodland (69.59% of the area) during the period. Construction land (land used for development) had decreased by 991 km^2^, a decrease rate of 59.84% from 1995 to 2000, and increased to 2349 km^2^, an increase rate of 71.69% from 2000 to 2020. The proportion of grasslands and areas with water were negligible and overall, did not vary significantly in size over the study period. The main feature of land use change in the study area was the loss of grasslands and woodlands through development. The habitat quality model indicated that habitat quality was highest and degradation was lowest in Dayao mountain, Guxiu town, Qichong village and Beituo town. Habitat quality improved in Daguishan and Luokeng areas. Habitat quality was good in Daping mountain and Linzhouding, but they were highly fragmented with patches of low‐quality habitat of varying sizes. Habitats were severely degraded in the Dateng Gorge area. The rate of habitat degradation has slowed over time in the study area, but gradually increased in degradation intensity, and low‐quality habitats were widely distributed and overlapped with the crocodile lizards distribution area. We recommend that protected areas for the crocodile lizard be more closely monitored and managed to halt further decline in habitat quality.

## Graphical abstract

In this article, we showed the struggle between humankind and endangered species. We analyzed the impact of land‐use changes on crocodile lizard habitat quality based on the presence‐only data and multi‐temporal land‐use data (1995, 2000, 2010, 2015 and 2020) by the InVEST model (Integrated Valuation of Ecosystem Services and Trade‐offs). We found that the low quality areas were widely distributed and overlapped with the Crocodile lizard’s habitat. From 1995 to 2020, the degradation rate of habitat quality in the whole study area slowed down, but the intensity increased gradually. These results were consistent with our actual survey, implying the importance of Crocodile lizards habitat conservation.

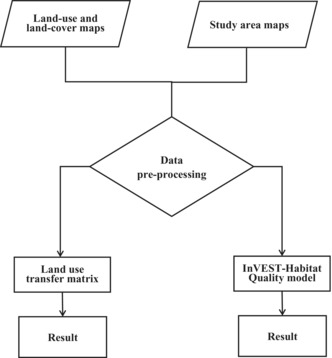

## 1 INTRODUCTION

Habitat quality refers to the ability of an ecosystem in a specific time and space to provide suitable and sustainable environments for organisms (Regolin et al., 2021). Habitat quality and availability can be used as proxies for biodiversity (Sharp et al., 2018). Understanding the spatiotemporal variability of habitat quality is important for expanding ecological conservation of wildlife (e.g., protect genetic diversity, predict population dynamics) (Crawford & Nusha, 2018; Thornton et al., 2013). In general, habitat quality varies with the intensity of nearby land use (Liu et al., 2022). Land use types, intensities and patterns alter the condition of natural resources and thus affect the survival and reproduction of wildlife (Dai et al., 2019; Whittington et al., 2019). Biogeochemical cycles and habitat quality for animals and plants are changed because of increased human disturbance (Abbott et al., 2019; Kiskaddon et al., 2019; Lin et al., 2020; Powers & Jetz, 2019). And changes in these cycling processes may have adverse effects on the structure and function of ecosystems. With urban expansion and development of land in developing countries, habitat quality is increasingly influenced at the landscape level, which has made habitat conservation to be an urgent issue (Liu et al., 2022).

Urbanization and industrialization have accelerated since the 20th century, and the over‐exploitation of land resources poses a severe threat to biodiversity (Deng et al., 2021). Because over‐exploitation of land can result in habitat degradation, fragmentation and loss (Brudvig et al., 2015). Several studies have concluded that land‐use and land‐cover changes (LULCC) activities are intensifying, and that wildlife habitat is increasingly being developed for agriculture and infrastructure (Jha & Bawa, 2006; Karki et al., 2018; Khan et al., 2021; Newbold et al., 2015). Evidence from different taxa and geographical regions suggested that land‐use was not equally affected all organisms in terrestrial ecological communities and that different functional groups of species may respond differently (Felipe‐Lucia et al., 2020; Newbold et al., 2020). The Researchers expected large carnivore populations to decline more in disturbed land than other animal groups (Newbold et al., 2020). However, amphibians and reptiles are the two most vulnerable groups of terrestrial vertebrates, being at a significantly higher risk than mammals and birds for threats such as habitat loss and fragmentation (Mayani‐Parás et al., 2019). Amphibians and reptiles generally have low dispersal abilities and are more habitat specialists than other vertebrates, making them particularly sensitive to landscape changes (Audrey et al., 2016; Joly et al., 2003; Wang et al., 2020). Therefore, habitat degradation and destruction are the focus of amphibian conservation. Despite many related studies have been conducted in mammals, birds, amphibians, it is amazing that little attention has been paid on reptiles (Gibbons et al., 2000) and are likely to be at a high risk of extinction (IUCN, 2006). The destruction and fragmentation of habitats reduce the structural complexity and functional integrity of habitats occupied by reptiles (Liu et al., 2016). Several factors, such as habitat loss, water pollution, climate change and mining, have been identified as negatively affecting breeding activities, reproduction and survival for reptiles (Becker et al., 2007; Gardner et al., 2007). These processes cause significant interference to the survival, reproduction and spread of reptiles, affecting species composition and community structure (Hung et al., 2017). Lately, these processes have also caused population declines due to the obstruction of population genetic exchange, reducing the range size of the species and resulting in local population extirpation (Mayani‐Parás et al., 2019).

Thus, effectively assessing and monitoring biodiversity and habitat quality changes and identifying the mechanisms causing these changes are essential for ecological management in fast‐changing and human‐dominated regions (Sun et al., 2019). There are three primary methods commonly used to evaluate changes in biodiversity and habitat quality: traditional field and habitat surveys (Do Nascimento et al., 2020), assessments of ecological indicators (Coates et al., 2016; Riedler & Lang, 2018) and simulations using ecological models (Akbari et al., 2021; Sallustio et al., 2017). Traditional terrestrial habitat monitoring methods are often time consuming, and their accuracy is difficult to assess due to differences between subjects (Lengyel et al., 2008). The Integrated Valuation of Ecosystem Services and Trade‐offs (InVEST) when used to evaluate biodiversity indicators or proxies of biodiversity, is a powerful tool to monitor biodiversity dynamics and habitat quality, especially in areas with limited available data on biodiversity (Sharp et al., 2016). Among the InVEST models, the habitat quality assessment model relies on the proximity of habitats to human land‐use and the intensity of land‐use (Sharp et al., 2018). Habitat quality is affected by habitat suitability, threats due to habitat quality reduction factors, habitat sensitivity to reduction factors and access to the habitat (Lee & Jeon, 2020). InVEST models introduced habitat quality as a proxy for biodiversity assessment (Gong et al., 2019). This approach allows for a rapid assessment of the status and changes in biodiversity status as a proxy for a more detailed biodiversity status (Sun et al., 2019).

The crocodile lizard (*Shinisaurus crocodilurus* Ahl, 1930) is a monotypic species in the monotypic family Shinisauridae. It is an ancient lineage from the Pleistocene, with ~100 million years of evolutionary history (Xie et al., 2021). Individuals of this species are diurnal, semiaquatic, viviparous and occur in rocky streams in cool mountain forests in southern China and northern Vietnam (Huang et al., 2008; van Schingen, Schepp, et al., 2015). The species is threatened with extinction due to continued deforestation, habitat destruction and poaching. As such, it is listed as endangered by the International Union for Conservation of Nature (IUCN) (Nguyen et al., 2014). Here, in order to fully analyze changes in habitat quality across the crocodile lizards distribution range, in conjunction with a habitat quality model, we set two main objectives: (1) analyzing land‐use change in the study area from 1995 to 2020 and (2) assessing habitat quality in the crocodile lizards distribution areas of Guangdong and Guangxi.

## 2 MATERIALS AND METHODS

### 2.1 Study area

**FIGURE 1:**
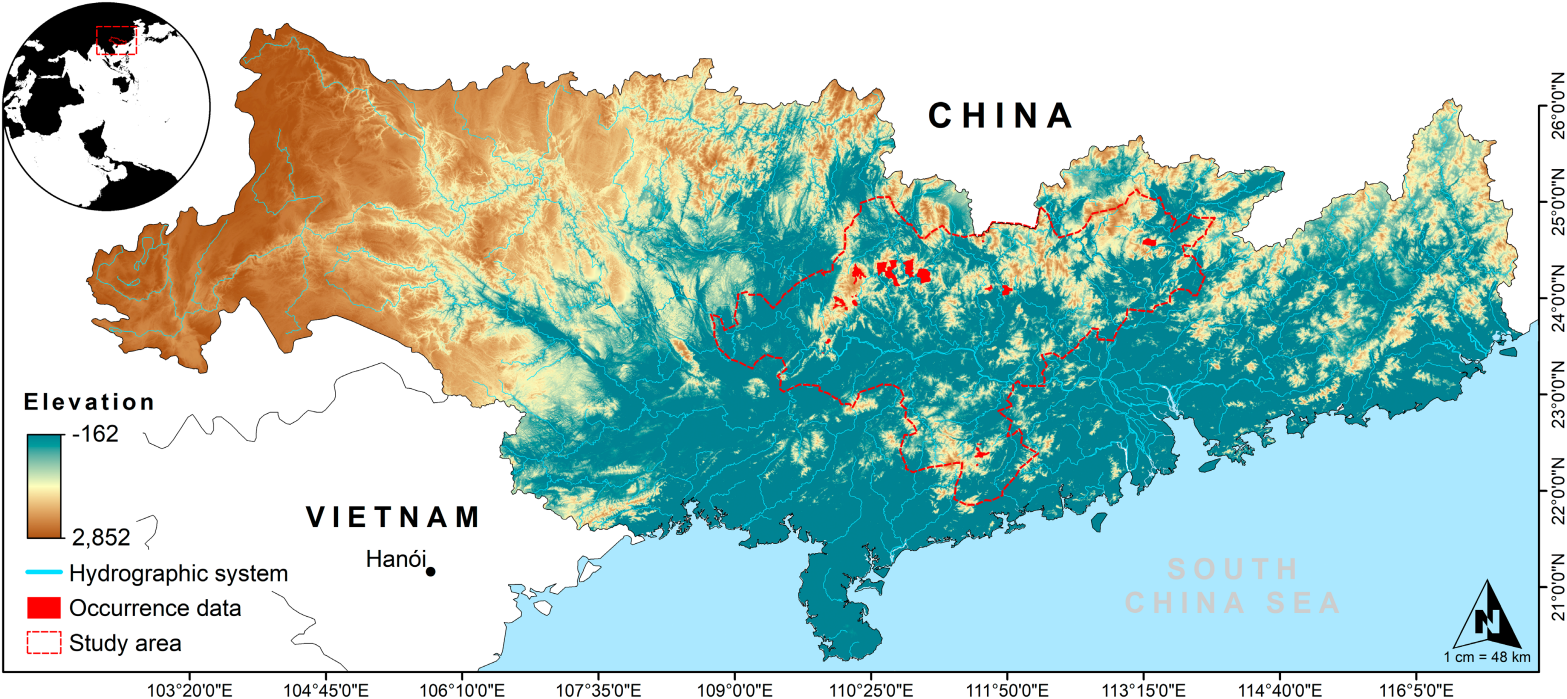
Geographic location of the study area, regional hydro‐topographic configuration and occurrence data for the target species.

The distribution range of crocodile lizards is restricted to southern China and northern Vietnam, where suitable habitat consists of small, isolated, fragmented and steadily shrinking habitat patches (Huang et al., 2008; Le & Ziegler, 2003; van Schingen, Ihlow, et al., 2014). Within Guangdong and Guangxi, populations are relatively scattered and far apart (Figure 1). Therefore, we selected part of the Pearl River Basin as the primary research area, including all crocodile lizards distribution areas (102°14′ to 115°53′ E, 21°31′ to 26°49′ N). This river spans the Yunnan‐Guizhou Plateau, the hills of Guangdong and Guangxi and the Pearl River Delta Plain from west to east (He et al., 2018; Wang et al., 2021). The climate in the study region has subtropical monsoon features, where the annual average temperature is approximately 14–21°C. And annual precipitation ranges between 1200 and 2200 mm (Wu et al., 2019), decreasing from southeast to northwest and primarily falling during April–September. The dominant vegetation is composed of evergreen forests (~65.3%), followed by cropland (~18.1%) (Wang et al., 2021). They are mainly distributed in the middle of the basin, which happens to be in the transitional areas of high‐to‐low elevations in Guangxi province (Wang et al., 2021).

### 2.2 Data collection

Land‐use and land‐cover maps from 1995, 2000, 2010, 2015 and 2020 (1 × 1 km) were used in this research. Data from the crocodile lizards' distribution area mainly include the Dayao mountain, the Guxiu area, the Mengshan area, the Qichong area, the Beituo area, the Daguishan area, the Luokeng area and the Maoming. Crocodile lizards have been reported from all of these areas (Huang et al., 2008; Zhang, 1991). County‐level administrative zoning map and protected area boundary data were analyzed. The county‐level administrative zoning map was obtained from the Ministry of Natural Resources of China (http://bzdt.ch.mnr.gov.cn). Land‐use and land‐cover maps came from the Resource and Environmental Science Data Center of the Chinese Academy of Sciences (http://www.resdc.cn).

### 2.3 Land use transfer matrix

Land use data were classified in three levels, according to the “China Land Use/Land Cover Remote Sensing Monitoring Data Classification System” (https://www.resdc.cn/). We reclassified landscape types into 14 different types (Table 1). Then, we overlaid land use data from 1995, 2000, 2005, 2010, 2015 and 2020 to construct the land‐use transfer matrix, input/output direction and the area of each type of land‐use within the study area using the spatial analysis tools in ArcGIS10.6 (ESRI, America).

**TABLE 1:** Classification system of land‐use in study area.

Code number	Land‐use types	Code number	Land‐use types
1	Arable land	41	Canals
3	Grasslands	43	Reservoir ponds
6	Unused lands	45	Tidal flats
21	Woodlands	46	Beaches
22	Bush forests	51	Urban lands
23	Sparse woodlands	52	Rural settlements
24	Other woodlands	53	Construction land

### 2.4 InVEST‐Habitat quality model

The InVEST model allows for the calculation of habitat quality by combining the sensitivity of landscape type and the intensity of external threats by assessing the service function of biodiversity based on habitat quality (Peng et al., 2018). In ecology, the InVEST model has been successfully used to assess land‐use change and regional habitat quality. Plant ecology, animal ecology or bird ecology studies tend to target specific species and populations in target regions, assessing the habitat quality of biodiversity service functions (Bhagabati et al., 2014). Habitat quality was determined by a function using four factors: (1) the relative impact of each threat, (2) the relative sensitivity of each habitat type to each threat, (3) the distance between habitats and (4) sources of threats (Chen et al., 2016). At the pixel scale, the threat level of each pixel cell was translated into habitat quality using the total threat level and a half‐saturation function. The formula we used follows (Sharp et al., 2014):

(1) $$Q\mathrm{xj}=\mathrm{Hj}\times \left[1‐\left(\frac{{\mathrm{Dxj}}^{z}}{{\mathrm{Dxj}}^{z}+k^{z}}\right)\right]$$

where *Q*_xj_ is ecological habitat quality value of land use type j, H_j_ is a habitat quality score ranging from 0 to 1, where non‐habitat land‐use types are given by a score of 0, and perfect habitat classes score 1. In our study, H_j_ is the habitat suitability in Table 3. *k* is the half‐saturation constant (Liang & Liu, 2017; Sun et al., 2015) and z is a constant.

(2) $$D_{\mathrm{xj}}=\sum_{r=1}^{R}\sum_{y=1}^{Y_{r}}\left(\omega_{r}/\sum_{r=1}^{R}\omega_{r}\right)r_{y}i_{\mathrm{rxy}}\beta_{x}S_{\mathrm{jr}}$$

where $D_{\mathrm{xj}}$ represents the total threat level of the grid x in LULC or habitat type j, y indexes all grid cells on *r*'s raster map and $Y_{r}$ indicates the set of grid cells on the raster map of r. Note that each threat map can have a unique number of grid cells due to variation in raster resolution. $\omega_{r}$ is the weight; $r_{y}$ is the number of stress factors on the grid unit; $\beta_{x}$ is the accessibility level of grid x; $S_{\mathrm{jr}}$ is the sensitivity of landscape j to stress factors, ranging from 0 to 1; $i_{\mathrm{rxy}}$ is the stress factor influence distance. If $S_{\mathrm{jr}}=0$ then $D_{\mathrm{xj}}$ is not a function of threat r. In our study, $S_{\mathrm{jr}}$ is the sensitivity of different land use types to different ecological threat factors in Table 3. Also, note that threat weights are normalized so that the sum across all threat weights equals 1. The impact of threat r that originates in a grid cell y, $r_{y}$ on habitat in a grid cell x is given by $i_{\mathrm{rxy}}$. It is represented by the following equations, mainly including the linear or exponential distance‐decay function:

(3) $$i_{\mathrm{rxy}}=1-\left(\frac{d_{\mathrm{xy}}}{d_{r\max}}\right)\;\text{if}\;\text{linear}$$

(4) $$i_{\mathrm{rxy}}=\exp\left(-\left(\frac{2.99}{d_{r\max}}\right)d_{\mathrm{xy}}\right)\;\text{if}\;\text{exponential}$$

where $d_{\mathrm{xy}}$ is the linear distance between grid cells x, y and $d_{r\max}$ is the maximum effective distance of the reach across the threat space. Generally, the impact of a threat on a habitat decreases as the distance from the degradation source increases, so that grid cells that are more proximate to threats will experience higher impacts (Sharp et al., 2014).

We referred to InVEST model manual and related research, combined with the actual situation of the study area and crocodile lizards distribution areas (Huang et al., 2008), to determine the relevant parameter values (Sharp et al., 2014). We considered arable land, reservoirs, urban land, rural settlements and construction land as the main ecological threats to crocodile lizard habitat quality (Table 2). The ecological threats are weighted, reflecting the intensity of interference with the habitat types. We set the maximum range of action of each stressor, which means that the interference intensity of the stressor to the habitat types decreases with increasing distance. At the same time, we chose the attenuation function to describe the mode of threat mitigation in space. We assigned a value to the sensitivity of these threat factors (Table 3)—the higher the value, the more sensitive it is to ecological threats.

**TABLE 2:** Stress factors of the study area with their corresponding weight values, impact distances and types of response.

Stress factors	Maximum impact distance/km	Weight	Decay type
Arable land	8	0.8	Exponential
Reservoir	3	0.5	Exponential
Urban land	6	0.75	Exponential
Rural settlements	10	1	Exponential
Other construction land	1	0.4	Linear

**TABLE 3:** Sensitivity of different land use types to different ecological threat factors.

Code	Land use type	Habitat suitability	Ecological threat factors				
			Arable land	Reservoir	Urban land	Rural settlements	Other construction land
0	No data	0	0	0	0	0	0
1	Agricultural lands	0	0	0	0	0	0
3	Grassland	0	0	0	0	0	0
6	Unused land	0	0	0	0	0	0
21	Woodland	0.8	0.8	0.5	0.7	0.7	0.5
22	Bush forest	1	0.9	0.6	0.7	0.8	0.6
23	Sparse woodland	0.6	0.5	0.6	0.6	0.8	0.4
24	Other woodland	0.1	0.3	0.4	0.2	0.2	0.3
41	Canal	1	0.9	0.9	0.7	0.7	0.6
43	Reservoir pond	0	0	0	0	0	0
45	Tidal flat	0	0	0	0	0	0
46	Beach	0	0	0	0	0	0
51	Urban land	0	0	0	0	0	0
52	Rural settlement	0	0	0	0	0	0
53	Other construction land	0	0	0	0	0	0

### 2.5 Data processing

According to the guidance of the user manual (Sharp et al., 2014), rasterise land use data. All threats should be measured in the same scale and units (i.e. all measured in density terms or all measured in presence/absence terms) and not some combination of metrics (Sharp et al., 2014). Areas classified as “No Data” in the threat maps were reclassified. When the pixel did not contain a threat, we set the threat level for that pixel to zero (Sharp et al., 2014). According to the natural breakpoint method in ArcGIS software, the grid habitat quality of each study period was divided into four categories: poor (0–0.2), medium (0.2–0.5), good (0.5–0.7) and high (0.7–0.1) (Deng et al., 2021).

## 3 RESULTS

### 3.1 Land use change from 1995 to 2020

**FIGURE 2:**
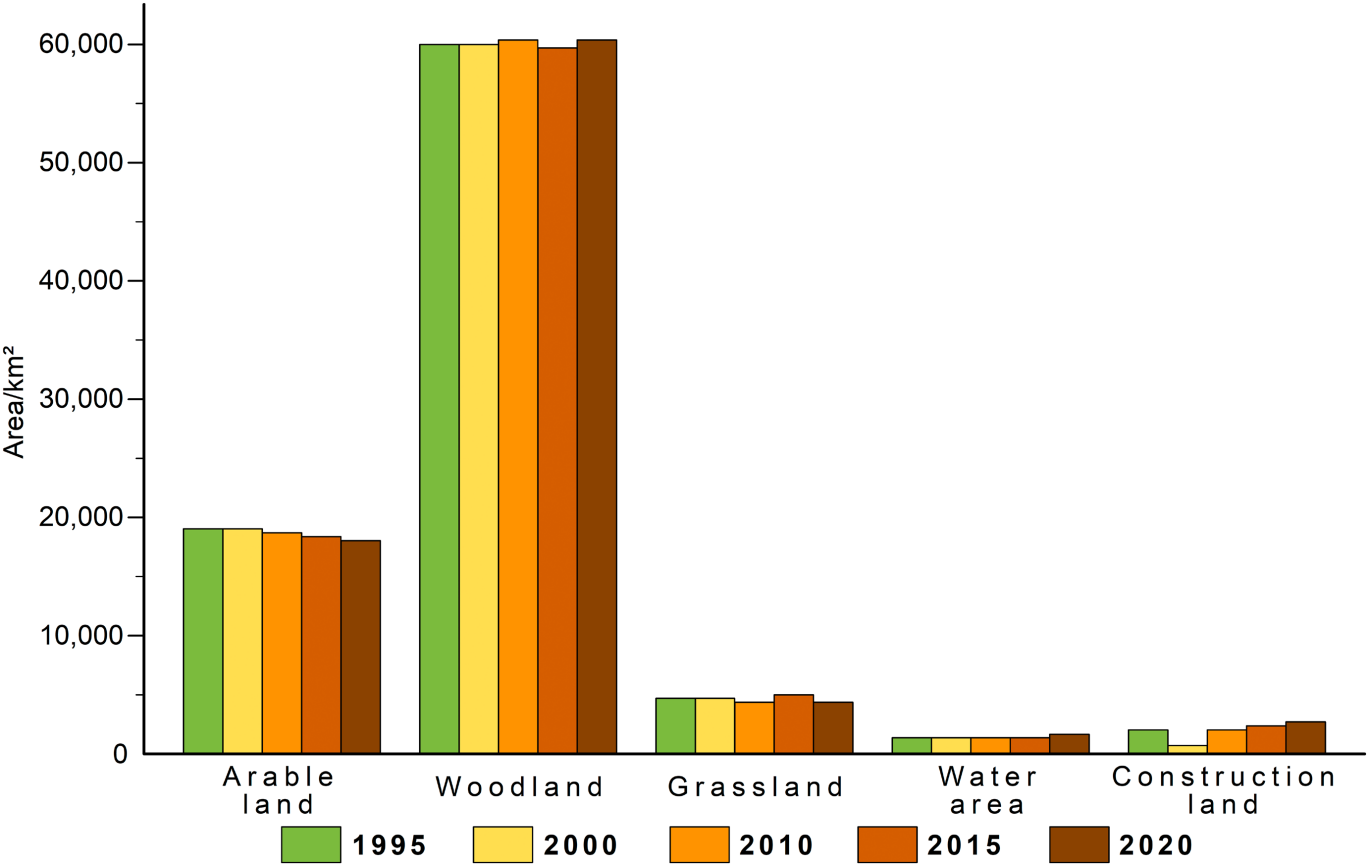
Annual surface variation, from 1995 to 2020, of the different land uses within the study area.

Our results showed that the leading landscape types in the study area were arable lands and woodlands in the past 25 years. The largest proportion of the area was woodland, which accounts for approximately 69.59% of the total study area, while arable land accounted for approximately 21.21%. Regarding the area change of each land‐use type, the arable lands acreage showed a downward trend decreasing from 18,405 km^2^ to 17,864 km^2^ between 1995 and 2020. Although the arable areas steadily decreased, the woodlands areas in the region remained relatively stable over time, covering near 60,233.60 km^2^. Construction land (land used for development) areas had decreased by 991 km^2^, a decrease rate of 59.84% from 1995 to 2000 and increased to 2349 km^2^, an increase rate of 71.69% from 2000 to 2020. The proportion of grasslands area and water areas was negligible and overall they did not vary a lot in size over the study period (Figure 2).

The land‐use transfer matrix showed that arable land and grassland gained land converted from woodland, as well as conversely woodland gained land converted from arable land and grassland. At the same time, construction land was growing in a faster way, with construction land gaining land converted from grassland and woodland during the study period (Table 4). From 1995 to 2020, 671,000 hm^2^ of arable land was converted to woodland, accounting for 68.41% of the area transferred from woodland. 655,000 hm^2^ of woodlands was converted into arable lands, accounting for 75.22% of the area transferred from arable land. 225,100 hm^2^ of grassland was converted into woodlands, accounting for 22.95% of the area transferred from grasslands. 12,600 hm^2^ of grasslands and 63,900 hm^2^ of woodlands were converted into construction land, accounting for 6.19% and 29.04% of the total area converted to construction land, respectively.

**TABLE 4:** The land‐use transfer matrix from 1995 to 2020s (hm^**2**^).

Period	Land type	Arable land	Woodland	Grassland	Water area	Construction land	Unused land	Total
2000								
1995	Arable land	1,837,400	500	100	1600	900	0	1,840,500
	Woodland	2300	6,025,200	2000	500	0	0	6,030,000
	Grassland	100	4100	501,400	100	0	0	505,700
	water land	100	0	0	136,900	0	0	137,000
	Construction land	0	0	0	100	165,500	0	165,600
	Unused land	0	0	100	0	100	300	500
	Total	1,839,900	6,029,800	503,600	139,200	166,500	300	8,679,300
2010								
2000	Arable land	1,826,000	4900	0	2300	6700	0	1,839,900
	Woodland	200	6,022,400	1100	1700	4300	100	6,029,800
	Grassland	0	11,800	491,100	100	600	0	503,600
	water land	200	400	0	138,500	100	0	139,200
	Construction land	100	200	0	0	166,200	0	166,500
	Unused land	0	0	0	0	0	300	300
	Total	1,826,500	6,039,700	492,200	142,600	177,900	400	8,679,300
2015								
2010	Arable land	1,807,700	300	100	600	17,800	0	1,826,500
	Woodland	300	5,992,600	34,400	200	12,300	0	6,039,800
	Grassland	0	0	490,000	0	2200	0	492,200
	water land	0	0	100	142,000	500	0	142,600
	Construction land	100	0	0	0	177,800	0	177,900
	Unused land	0	0	0	0	0	400	400
	Total	1,808,100	5,992,900	524,600	142,800	210,600	400	8,679,400
2020								
2015	Arable land	904,100	664,900	88,700	43,300	106,900	200	1,808,100
	Woodland	654,000	5,005,400	218,500	55,700	59,100	100	5,992,800
	Grassland	88,500	244,100	172,000	8500	11,400	100	524,600
	water land	42,100	52,100	7900	31,800	8900	0	142,800
	Construction land	97,300	47,900	7100	9700	48,600	0	210,600
	Unused land	300	100	0	0	0	0	400
	Total	1,786,300	6,014,500	494,200	149,000	234,900	400	8,679,300
2020								
1995	Arable land	915,500	671,000	89,600	46,000	118,200	200	1,840,500
	Woodland	655,000	5,033,500	220,500	56,900	63,900	100	6,029,900
	Grassland	89,000	225,100	170,500	8400	12,600	100	505,700
	water land	40,400	50,100	7600	30,100	8800	0	137,000
	Construction land	86,200	34,400	6000	7600	31,400	0l	165,600
	Unused land	200	300	0	0	0	0	500
	Total	1,786,300	6,014,400	494,200	149,000	234,900	400	8,679,200

Calculated in stages, the largest land‐use type was woodland in the study area between 1995 and 2000. Woodland significantly increased by gaining land converted from other land use types, mainly from grassland (4100 hm^2^) and arable land (500 hm^2^), accounting for 93.35% of the area converted from grassland and 16.13% of the area converted out from cropland, respectively. From 2000 to 2010, construction land, woodland and areas with water sources became the main land‐use types. Woodland has gained access to land converted from other land use types, mainly from arable lands (4900 hm^2^) and grasslands (11,800 hm^2^). Construction land (11,000 hm^2^) and water areas (4000 hm^2^) all gained land converted from other land use types, mainly from arable land and woodlands. From 2010 to 2020, 124,700 hm^2^ of arable land and 71,400 hm^2^ of woodland was converted to construction land, increasing nearly 18‐fold compared with the areas converted to construction land in the past 10 years.

### 3.2 Temporal and spatial dynamics of habitat quality

#### 3.2.1 Habitat quality in the Pearl River Basin

**FIGURE 3:**
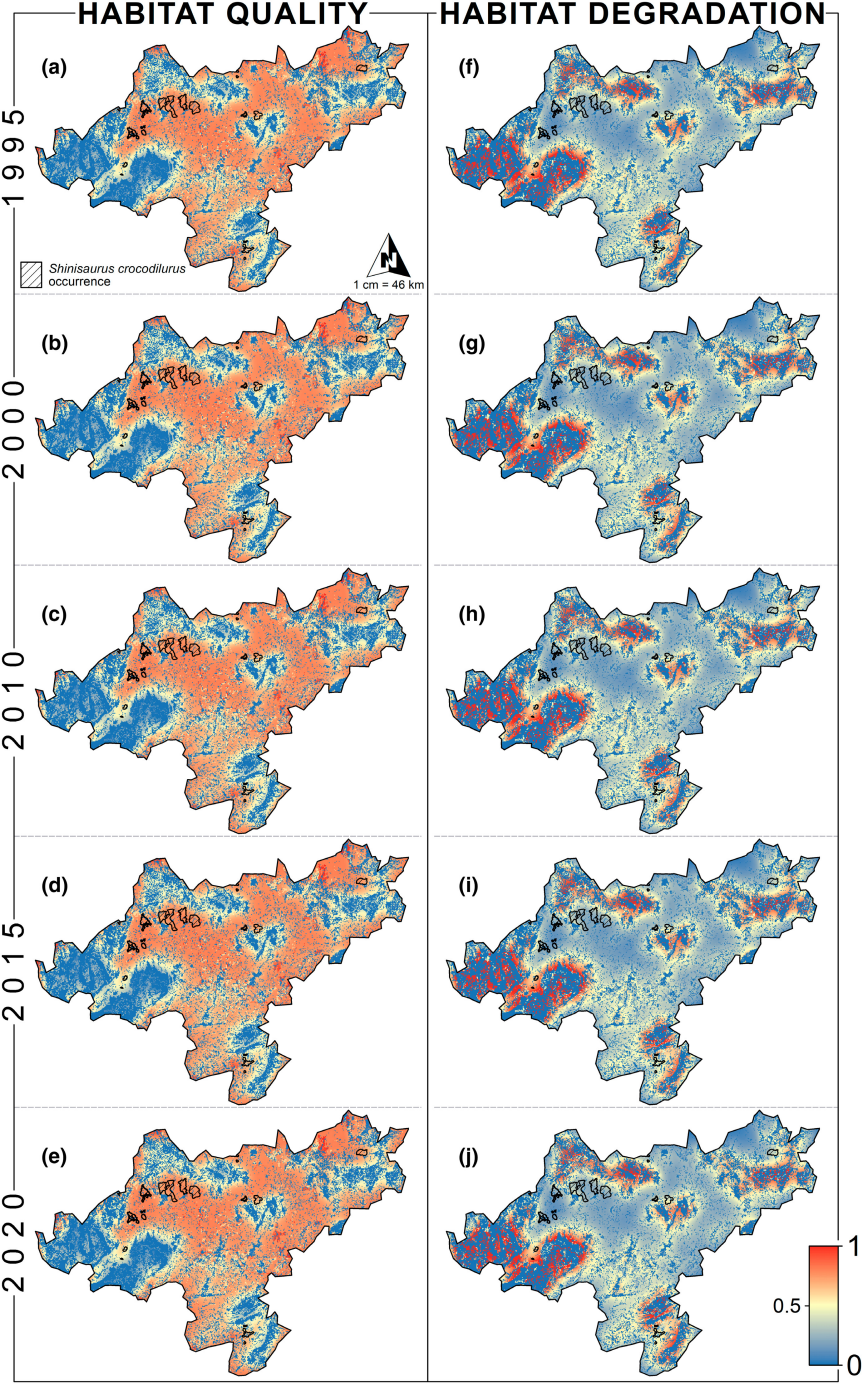
Spatial–temporal distribution characteristics of habitat quality and habitat degradation from 1995 to 2020. For habitat quality, red indicates high habitat quality and blue indicates low habitat quality; for habitat degradation, red indicates severe habitat degradation and blue indicates weak habitat degradation.

Based on the habitat quality calculations (Table 5), the habitat quality in the study area showed a “decrease–increase” trend from 1995 to 2020, consistent with the results of the land‐use transfer matrix. The standard deviation of the habitat quality index increased from 0.3218 to 0.3250 between 1995 and 2015 (Table 5). The maximum habitat degradation degree decreased from 0.1301 to 0.1285 from 1995 to 2015. Nevertheless, the maximum of the habitat degradation degree increased to 0.1332 after 2015 (Table 5). The habitat quality model showed that habitat quality within the study area did not vary significantly over time scales. Low habitat quality areas were widely distributed, mainly concentrated in counties and districts around the crocodile lizards' range (Figure 3).

**TABLE 5:** Spatial statistics of habitat quality and degradation in the study area.

Year	Statistical parameters of habitat quality				Statistical parameters of habitat degradation degree			
	Minimum	Maximum	Average	Standard deviation	Minimum	Maximum	Average	Standard deviation
1995	0	0.999	0.4505	0.3218	0	0.1301	0.0218	0.0205
2000	0	0.999	0.4487	0.3221	0	0.1301	0.0217	0.0205
2010	0	0.999	0.4431	0.3235	0	0.1299	0.0214	0.0203
2015	0	0.999	0.4408	0.3250	0	0.1285	0.0211	0.0202
2020	0	0.998	0.4458	0.3248	0	0.1332	0.0211	0.0199

#### 3.2.2 Habitat quality of *Shinisaurus crocodilurus* distribution area

We calculated the habitat quality index of the crocodile lizards distribution area separately (Table 6). The habitat quality index of the crocodile lizards' distribution area was consistent with the results of the whole study area, which first decreased and then increased. The degree of habitat degradation was different from that of the entire study area. The mean value of the habitat degradation degree declined from 1.9353 to 1.9060 between 1995 and 2015. After that, the mean value of the habitat degradation degree increased to 1.9356 and the maximum value decreased from 5.8231 to 5.5984.

**TABLE 6:** Spatial statistics of habitat quality and degradation in the crocodile lizards' distribution area.

Year	Statistical parameters of habitat quality				Statistical parameters of habitat degradation degree			
	Minimum	Maximum	Average	Standard deviation	Minimum	Maximum	Average	Standard deviation
1995	0	0.9665	0.6838	0.1971	0	5.8231	1.9353	0.8633
2000	0	0.9665	0.6816	0.2004	0	5.8223	1.9292	0.8627
2010	0	0.9659	0.6795	0.2030	0	5.8058	1.9267	0.8638
2015	0	0.9737	0.6798	0.2077	0	5.7627	1.9060	0.8702
2020	0	0.9658	0.6814	0.2029	0	5.5984	1.9356	0.8832

**FIGURE 4:**
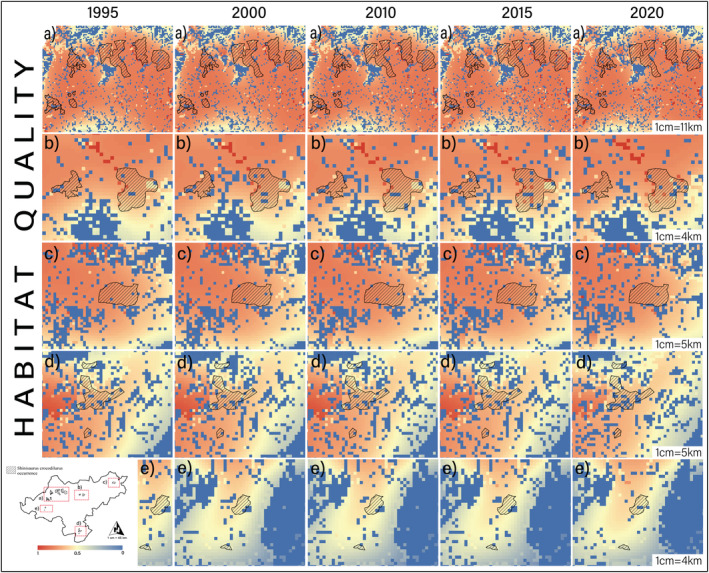
Spatial–temporal distribution characteristics of habitat quality from 1995 to 2020. (a) Dayao Mountain (DYS), Guxiu area (GX), Mengshan area (MS), Qichong area (QC), Beituo area (BT); (b) Daguishan area (DGS); (c) Luokeng area (LK); (d) Linzhouding area (LZD); (e) Daping mountain (DPS, above), Dateng gorge area (DTX, below). Red indicates high habitat quality and blue indicates low habitat quality.

**FIGURE 5:**
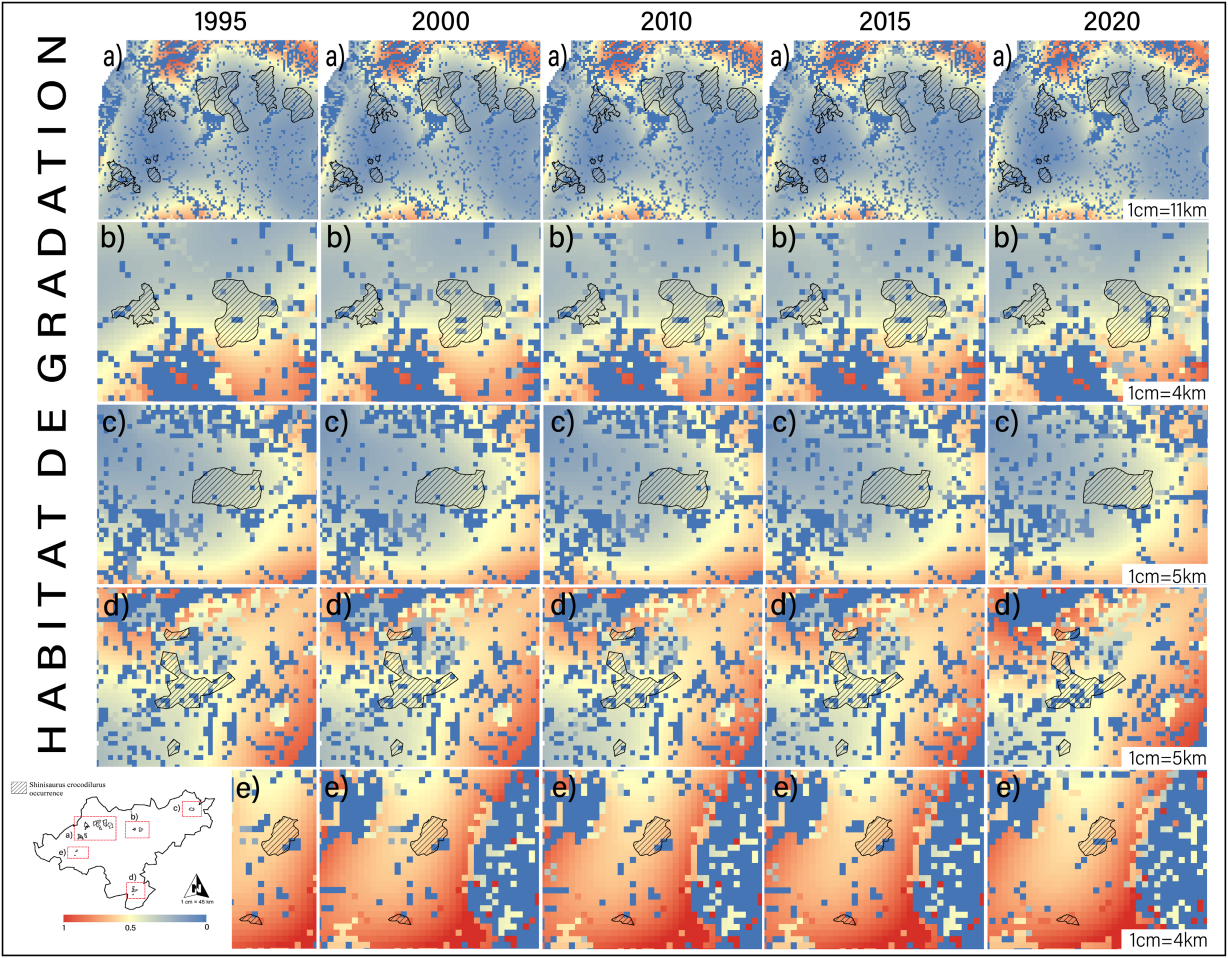
Spatial–temporal distribution characteristics of habitat degradation from 1995 to 2020. (a) Dayao Mountain (DYS), Guxiu area (GX), Mengshan area (MS), Qichong area (QC), Beituo area (BT); (b) Daguishan area (DGS); (c) Luokeng area (LK); (d) Linzhouding area (LZD); (e) Daping mountain (DPS, above), Dateng gorge area (DTX, below). Red indicates severe habitat degradation and blue indicates weak habitat degradation.

Spatial distribution of habitat quality indicated that the Dayao Mountain (DYS), Guxiu, Qichong (GX) and Beituo areas (BT) had the highest habitat quality and the lowest degree of habitat degradation during the period (Figures 4 and 5). Subsequently, habitat quality in the Dagui Mountain (DGS) and Luokeng areas (LK) remains positive, with some areas of poor habitat quality. Habitat quality was better in Daping Mountain (DPS) and Linzhouding (LZD), but these patches were highly fragmented patches and low‐quality patches of varying sizes. The worst habitat quality was found in the Dateng Gorge area (DTX) and accompanied by large‐scale anthropogenic disturbance (Figure 5).

## 4 DISCUSSION

### 4.1 The impact of land‐use change on the crocodile lizards' habitat

In our study, the land‐use transition matrix was used to explore the temporal and spatial changes in land‐use types in the lizards' distribution range. We found that the main landscape types in the study area were arable land and woodland during the period 1995 to 2020. Over time, the construction land shows a “decrease–increase”, especially from 2000 to 2020, during which construction land area peaked at 71.69%. From 2010 to 2020, a large amount of arable land and woodland was used for economic development or rural residences. Therefore, it was probable that the study area has experienced rapid economic development and urbanization in the past 25 years (Zhang et al., 2018). It is worth noting that the change in the range of the crocodile lizards fitted with the pattern of land‐use change in the study area. In other words, as the area of construction land increased, the crocodile lizards' distributions range gradually decreased. These results suggested that land‐use change had a negative impact on the habitat of the crocodile lizards. A survey showed that none of the previously reported crocodile lizards were found in Xiali and Beituo of Mengshan County and Xianhui of Zhaoping County in Guangxi, and the crocodile lizards in these areas may have become extinct (Huang et al., 2008). At the same time, suitable habitat is steadily shrinking due to illegal logging and coal mining (van Schingen et al., 2016). Species distribution models showed that potentially suitable habitat for crocodile lizards is fragmented, small and disconnected with extremely poor coverage within protected areas (van Schingen, Ihlow, et al., 2014). The negative impact was also evident in the Yangtze River basin, where wildlife habitat degradation has increased in the middle and lower reaches of the Yangtze (Li et al., 2021). The negative impacts of urbanization on habitat quality have surpassed the positive effects of environmental protection programs (Li et al., 2021). Established areas extended further into natural habitats (Haase et al., 2014; Hennig et al. 2015), and such encroachment may ultimately affect conservation hotspots, even if they are located far from urban centers (McDonald et al., 2014). Therefore, in order to prevent the ecological disaster caused by the loss of biodiversity, the implementation of environmental protection policies, along with environmental conservation and restoration programs, must be rigid (Li et al., 2021).

### 4.2 Habitat quality change of crocodile lizards

The InVEST model showed that the low‐quality habitats were widely distributed, mainly in the periphery of the crocodile lizard's distribution areas. High‐quality habitats were concentrated in the mountainous forest areas in the central and eastern part of the study area, mainly DYS, GX, QC, BT, DGS and LK. Our study was consistent with previous fieldwork investigations. The long evolutionary history of crocodile lizards as well as their life history traits make them highly sensitive to environmental conditions (Wu et al., 2012; Ziegler et al., 2019). Crocodile lizards are “living fossils”, and the only surviving member of their family (Xie et al., 2021). The ecological niche of crocodile lizards are in valleys below 800 m.a.s.l. and appears to be restricted to tiny sections of clean and remote streams (van Schingen, Pham, et al., 2015; Wu et al., 2007; Zhao et al., 2006; Ziegler et al., 2019). High habitat quality areas, such as DYS, GX, QC and BT are mainly located in sparsely populated mountainous areas (e.g. within Jinxiu county, Mengshan county and Zhaoping county). During the Cenozoic era, Dayao Mountain (DYS) was located in the central region of the Guangxi Arcuate Mountains, an essential pathway for animal migration in the Guangxi province (Huang et al., 2014), where the terrain was high in the middle, before dropping off, and the climate was warm and rainy. Based on genetic analyses and population demography of crocodile lizards, Dayao Mountain (DYS) may be an ancient refuge for this species in the history (Huang et al., 2014). Guangxi and Luokeng might have been the source of an initial population expansion (Huang et al., 2014). Initial field surveys showed that between 1977 and 1991, the main distribution sites of crocodile lizards in Guangxi were within DYS, BT, DGS, QC, GX and MS (Zhang, 1991; Zhang et al., 2005). From 2001 to 2004, the main distribution sites of crocodile lizards in Guangxi decreased, with crocodile lizards present in the wild mainly in DYS, BT and DGS (Zhang et al., 2005). Field surveys in 2008 showed that none of the previously reported crocodile lizards were found in BT of Mengshan County in Guangxi, and the crocodile lizards in these areas may have become extinct (Huang et al., 2008). Poaching and habitat fragmentation may be responsible for the result (Huang et al., 2008; Ziegler et al., 2019). At the same time, crocodile lizards have high requirements for water quality in their habitat. In Vietnam, streams inhabited by crocodile lizards are characterized as soft waters (GH < 1–2) (where GH = general hardness) with a high‐water quality, indicated by a high oxygen content, and low nutrient concentrations of nitrogen and phosphate (van Schingen, Pham, et al., 2014; Ziegler et al., 2019). Furthermore, the water ranges from neutral to relatively acidic conditions with pH values ranging from 4.5 to 7.37, while pH values of 6.5 were measured in Dayaoshan Nature Reserve, Guangxi, China (DYS; Long et al., 2007; van Schingen, Pham, et al., 2014). And crocodile lizards prefer habitats with sandy water substrates because the abundance of sand in the water body provides a buffering effect and also enables crocodile lizards to climb from out of the water to land (Wu et al., 2012). Thus, the high‐quality habitats located in mountainous forest areas have not been subject to significant anthropogenic disturbance for the time being, which provides conducive areas for the continued reproduction of the crocodile lizards.

Our results showed that the habitat quality in the Dateng Gorge has been poor in the past 25 years, with high levels of habitat degradation. This might be connected with the construction of water conservancy and hydropower projects. The Dateng Gorge is a canyon in the lower reaches of the Qianjiang River in the West River system of the Pearl River Basin, formed by the Qianjiang waterway between the Dayao Mountains and the Lotus Mountains (Yang et al., 2017). The connection of the mountains may provide a migration channel for the crocodile lizards, which may be a fundamental reason for its presence (Yang et al., 2017). Upon completion of the Dateng Gorge Water Conservancy Project, the downstream area of the ditch in the Dawandu sub‐field where crocodile lizards had been recorded, especially the creeks where crocodile lizards are widely distributed, will be submerged to the middle reaches (Yang et al., 2017). Hydropower facilities fragment streams into several channel segments and can alter the flow and sediment regimes (Csiki & Rhoads, 2014; Fantin‐Cruz et al., 2015; Takahashi & Nakamura, 2011) and inhibit the dispersal of riparian plants and the migration of aquatic organisms (Andrea et al., 2012; Chen et al., 2015; Fencl et al., 2015; Perkin et al., 2015; Zhang et al., 2021). The situation has resulted in the loss of better quality habitat for crocodile lizards or even a break in flow, which had a significant negative impact on the growth and development of crocodile lizards (Yang et al., 2017). Moreover, during the construction of mining roads, large amounts of blasting and excavation debris were dumped into the stream, causing the pollution of inhabited streams (van Schingen, Pham, et al., 2014; van Schingen, Schepp, et al., 2015; Yu et al., 2005). And local villagers often use electro‐fishing and poisonous chemicals to fish in the stream and this can kill all of the crocodile lizards in the water (Huang et al., 2008), further exacerbating the decline of wild populations and loss of habitat. Thus, future economic development in the Dateng Gorge area should be minimized in order to protect the current limited habitat of the crocodile lizards.

### 4.3 Conservation suggestions

In the face of a massive crisis of deteriorating habitat quality for the lizards, while coping with local habitat destruction due to agricultural purposes, agreements with respective local farms helped to keep at least core zones of important habitats intact in the crocodile lizards' nature reserve in China (van Schingen, Schepp, et al., 2015). Second, the Chinese government should encourage the development of the local economy and educate local people about the laws relating to wildlife conservation and prohibit the capture or trade of crocodile lizards. Third, the nature reserves should be expanded to restore forest conditions within the reserves to create more suitable habitats for the crocodile lizard. Further, Chinese crocodile lizards could be bred in captivity in nature reserves and released back into nature to restore the wild populations (Huang et al., 2008). Therefore, a breeding station was constructed in 2003, and the first round of crocodile lizards released back into the wild (Long et al., 2007; Zollweg, 2011, 2012). In 2009, 30 crocodile lizards were released into the Guangdong Luokeng Crocodile Lizard Provincial Nature Reserve (Zhong, 2009). Fifteen crocodile lizards were released into the wild for the first time in the Daguishan crocodile lizards National Nature Reserve in 2019 (Tang et al., 2019). The Department of Forestry of Guangxi Zhuang Autonmous Region released 20 crocodile lizards into the Daguishan crocodile lizards National Nature Reserve in September 2020 (Hu, 2020). The efforts have already led to a stable and even slightly increasing subpopulation within the Daguishan Nature Reserve in 2011 (Zollweg, 2012).

## 5 CONCLUSIONS

Based on the land‐use transfer matrix and the InVEST model, we analyzed the temporal and spatial dynamics of land‐use change trends and habitat quality in the crocodile lizard's distribution area in the Pearl River Basin from 1995 to 2020. The land use transfer matrix showed that the dominant landscape types were arable land and woodland in the study area. From 1995 to 2020, the arable land area decreased from 18,405 km^2^ to 17,864 km^2^, and the construction land area showed a “decrease – increase” trend. A large amount of arable land (118,200 km^2^) and woodland (63,900 km^2^) was used for economic development (58.08% compared with 31.40%), indicating that the study area had experienced rapid economic development and increasing urbanization in the past 25 years. The InVEST model showed that the low‐quality habitats were widely distributed, mainly in the periphery of the crocodile lizards distribution areas. High‐quality habitats were concentrated in the mountainous forest areas in the central and eastern part of the study area. Among the various crocodile lizard populations, habitat quality was highest and degradation was lowest in DYS, GX, QC and BT. Habitat quality was better in DGS and LK. Habitat quality was good in DPS and LZD, but they were highly fragmented with patches of low‐quality habitat of varying sizes. Habitat quality was poor and habitats were severely degraded in the Dateng Gorge. However, with rapid economic development, human footprint has gradually expanded into the remaining suitable habitat for crocodile lizards, with serious impacts on their habitats. Effective conservation of current crocodile lizard habitat and restoration of wild populations is urgently required. Changes in land‐use and landscape patterns are a visual indication of the effectiveness of conservation in nature reserves. There is conflict between conservation and development in nature reserves, and it is important to get the right relationship between them.

## AUTHOR CONTRIBUTIONS

**Xiaoli Zhang:** Conceptualization (equal); data curation (equal); formal analysis (equal); funding acquisition (equal); investigation (equal); methodology (equal); project administration (equal); resources (equal); software (equal); supervision (equal); validation (equal); visualization (equal); writing – original draft (equal); writing – review and editing (equal). **Dongxu Qin:** Conceptualization (equal); data curation (equal); formal analysis (equal); funding acquisition (equal); investigation (equal); methodology (equal); project administration (equal); resources (equal); software (equal); supervision (equal); validation (equal); visualization (equal); writing – original draft (equal); writing – review and editing (equal). **Facundo Alvarez:** Conceptualization (equal); data curation (equal); formal analysis (equal); funding acquisition (equal); investigation (equal); methodology (equal); project administration (equal); resources (equal); software (equal); supervision (equal); validation (equal); visualization (equal); writing – original draft (equal); writing – review and editing (equal). **Zening Chen:** Conceptualization (equal); data curation (equal); formal analysis (equal); funding acquisition (equal); investigation (equal); methodology (equal); project administration (equal); resources (equal); software (equal); supervision (equal); validation (equal); visualization (equal); writing – original draft (equal); writing – review and editing (equal). **Zhengjun Wu:** Conceptualization (equal); data curation (equal); formal analysis (equal); funding acquisition (equal); investigation (equal); methodology (equal); project administration (equal); resources (equal); software (equal); supervision (equal); validation (equal); visualization (equal); writing – original draft (equal); writing – review and editing (equal).

## FUNDING INFORMATION

This study was funded entirely by the National Natural Science Foundation of China (NSFC grant: 31760623,32160131), Biodiversity Survey, Monitoring and Assessment Project of Ministry of Ecology and Environment, China (2019HB2096001006) and Guangxi Natural Science Foundation, China (AD21220058), Project of Guangxi Daguishan National Nature Reserve and Project of Guangdong Luokeng National Nature Reserve.

## CONFLICT OF INTEREST

The authors declare that this research was conducted out with commercial and/or financial concerns and is free of potential conflicts of interest.

### OPEN RESEARCH BADGES

This article has earned Open Data and Open Materials badges. Data and materials are available at https://doi.org/10.5061/dryad.s1rn8pk9n.

## ETHICS STATEMENT

The animal study was reviewed and approved by Institutional Animal Care and Use Committee (IACUC) of Guangxi Normal University (Reference Number: 202205–001).

## Data Availability

https://doi.org/10.5061/dryad.s1rn8pk9n
